# Expression of NGF, proNGF, p75^NTR^ in lung injury induced by cerebral ischemia-reperfusion in young and elderly rats

**DOI:** 10.1016/j.clinsp.2024.100532

**Published:** 2024-11-16

**Authors:** Hong Chen, Qiang Du, Jie Chen, Qiang Tian, Lei Xu, Ying Wang, Xiaoyan Gu

**Affiliations:** a Center of Chinese Medicine Rehabilitation, The Second Affiliated Hospital of Nanjing University of Chinese Medicine, Nanjing City, Jiangsu Province, China; b Department of Respiratory and Critical Care Medicine, The Second Affiliated Hospital of Nanjing Medical University, Nanjing City, Jiangsu Province, China; c Department of Rehabilitation Medicine, Zhenjiang Hospital of Chinese Traditional and Western Medicine, Zhenjiang City, Jiangsu Province, China

**Keywords:** NGF, proNGF, p75^NTR^, Cerebral ischemia, Acute lung injury

## Abstract

**Objective:**

This study aims to investigate the expression levels of Nerve Growth Factor (NGF), the precursor form of NGF (proNGF), and p75 neurotrophin receptor (p75^NTR^) in lung injury induced by cerebral Ischemia-Reperfusion (I/R) in both young and elderly rats.

**Methods:**

Male Sprague-Dawley rats, categorized as young (3-months-old) and elderly (16-months-old), were divided into four experimental groups: Young Sham, Young I/R, Elderly Sham, and Elderly I/R. Each group underwent either sham surgery or ischemia-reperfusion treatment. Following 24 h post-procedure, the severity of cerebral ischemia was assessed using the Zea Longa 5-point scoring system, and lung tissue pathological changes were examined using Hematoxylin and Eosin (HE) staining. Western blot analysis was utilized to measure the expression levels of NGF, proNGF, and p75^NTR^ proteins in lung tissue.

**Results:**

Both young and elderly I/R groups exhibited lung tissue congestion and edema compared to their respective sham groups, with a significant increase in pathological scores (*p* < 0.05). Furthermore, the elderly I/R group demonstrated a significantly higher pathological score compared to the young I/R group (*p* < 0.05). Western blot analysis revealed that compared to the young sham group, the expression of NGF in the lung tissue of elderly sham rats decreased (*p* < 0.05), while proNGF and p75^NTR^ increased (*p* < 0.05). Additionally, compared to the sham group, the levels of NGF, proNGF, and p75^NTR^ in lung tissue were elevated in both young and elderly I/R groups of rats (*p* < 0.05). Moreover, the expression of proNGF and p75^NTR^ in lung tissue was higher in the elderly I/R group than in the young I/R group (*p* < 0.05).

**Conclusion:**

Cerebral ischemia-reperfusion-induced lung injury was associated with increased expression of proNGF and p75^NTR^, as well as decreased NGF expression in lung tissue. These alterations in NGF, proNGF, and p75^NTR^ may contribute to the susceptibility to age-related lung injury.

## Highlights

• A slightly increased vulnerability to lung injury with age, even without I/R.
• Assessment illustrates an increase in pathology scores with age and I/R treatment.
• Elucidates the alternation of NGF, proNGF, and p75NTR expression in lung tissue.
• These alterations may contribute to the susceptibility to age-related lung injury.

## Introduction

Ischemic stroke is prevalent among the elderly population. Currently, the rapid restoration of blood flow stands as the most effective treatment for ischemic stroke. However, Ischemia/Reperfusion (I/R) injury, resulting from cerebral hypoxia/reoxygenation and blood reperfusion, is inevitable.^1^ Recent studies indicate that mechanisms underlying brain I/R injury involve disruptions in energy metabolism, cellular acidosis, increased synthesis or release of excitotoxic amino acids, disturbances in intracellular calcium homeostasis, free radical production, and activation of apoptosis genes.^1^ Moreover, it can induce damage to distant vital organs, significantly impacting patient prognosis.^2^

Lung injury is the most frequent severe complication and cause of death in stroke patients.^3^ Brain injury triggers a robust activation of the sympathetic nervous system, leading to significant peripheral immunosuppression and an elevated risk of poststroke infection.^4^ Stroke-associated lung injury is characterized by pulmonary inflammation and impaired cellular immune function. Studies demonstrated increased levels of proinflammatory cytokines in bronchoalveolar lavage fluid from patients with severe cerebral damage.^5^ Animal research has further shown elevated concentrations of proinflammatory cytokines throughout the lungs following ischemic stroke, resulting in marked inflammation in bronchoalveolar lavage fluid and significant diffuse alveolar injury, along with pulmonary edema.^6^^,^^7^ Elderly individuals, due to immunosenescence and declining immune function, are particularly vulnerable to ischemia-reperfusion-induced lung injury.

While previous research has illuminated some aspects of lung injury resulting from cerebral ischemia-reperfusion, the detailed mechanisms regulating this process, particularly the roles of specific neurotrophic factors, remain poorly understood. Nerve Growth Factor (NGF), a crucial regulator of neuroimmune plasticity, is also implicated in respiratory system-related diseases.^8^ NGF typically promotes tissue repair and survival through its interaction with the TrkA receptor. However, its precursor form, proNGF, has a higher affinity for the p75 Neurotrophin Receptor (p75^NTR^), which is associated with apoptotic pathways. This difference in receptor affinity leads to distinct biological outcomes: NGF/TrkA interaction generally supports cell survival and tissue repair, while proNGF/p75^NTR^ interaction tends to promote apoptosis and inflammation.^9^

In the context of lung injury, elevated levels of proNGF and increased signaling through p75^NTR^ have been linked to heightened inflammatory responses and cellular apoptosis.^10^ This relationship suggests that the balance between NGF and proNGF, and their respective receptor interactions, may play a critical role in the development and severity of lung injury following cerebral ischemia-reperfusion. Specifically, in elderly individuals, the upregulation of proNGF and p75^NTR^ may exacerbate lung injury by amplifying inflammatory processes and promoting cell death in pulmonary tissues.^10^

This study aims to elucidate the impact of aging on Ischemia-Reperfusion (I/R)-induced lung injury by establishing rat models of brain artery I/R-induced lung injury in both young and elderly rats with equivalent neurofunctional damage. The present study's objective is to investigate the potential roles of NGF, proNGF, and p75^NTR^ in the heightened susceptibility of elderly brains to I/R-induced lung injury, thereby laying a theoretical foundation for the prevention and treatment of such injury in the elderly.

## Materials and methods

### Experimental animals and grouping

Young male Sprague-Dawley rats (3-months-old) and elderly male Sprague-Dawley rats (16-months-old) were obtained from Beijing Huafukang Biotechnology Co., LTD. Prior to and during the experiment, all rats were housed under controlled conditions with a 12-hour light/dark cycle at 22°‒25 °C and 40 %‒60 % humidity. Rats had free access to standard laboratory food and water ad libitum. The average weight of young rats was approximately 250 gs, and elderly rats weighed around 450 gs. This study followed the ARRIVE guidelines for animal research. The experimental protocol was approved by the Animal Experimentation Ethics Committee of Nanjing University of Chinese Medicine. The Ethics Committee study protocol number is 202309A008.

### Establishment of the rat model of cerebral I/R injury

Rats were fasted for 12 h prior to surgery but were allowed free access to water. Anesthesia was initiated and maintained with isoflurane, delivered at an induction dose of 5 % for 3 min, and maintained at 2 % during surgical procedures through a specialized rodent anesthesia system. In case of anesthesia failure, evidenced by physical movement or response to surgical manipulation, a secondary dose of isoflurane (2 % for 1 min) was administered for re-induction. After anesthesia, rats were fixed in the supine position, and the neck was routinely disinfected. The right common carotid artery and its branches were isolated, and the common carotid artery was occluded with an aneurysm clip. In the young I/R group, a silicone rubber head with a diameter of 0.32 mm was inserted into the right middle cerebral artery to a depth of (18 ± 2 mm). In the elderly I/R group, a silicone rubber head with a diameter of 0.43 mm was inserted into the right middle cerebral artery to a depth of (22 ± 2 mm). The silicone rubber heads were removed after 2 h, followed by hemostasis and suturing of muscle and skin layers. The young sham surgery group and elderly sham surgery group underwent neck carotid artery dissection without the insertion of silicone rubber heads. After surgery, animals were placed in cages with clean bedding, room temperature controlled at 22‒25 °C, and given free access to food and water. After 24 h, rats were evaluated using the Zea Longa 5-point scoring system,^11^ and rats with scores of 1‒3 were selected for the following experiment.

### HE-saining

The rats were anesthetized using isoflurane, positioned supinely, and euthanized promptly via decapitation. Whole lung extraction followed, and the right lung tissue was fixed with 4 % paraformaldehyde. Subsequently, they were examined under a light microscope (Nikon Eclipse 80i). Lung injury pathology was quantitatively assessed based on a standardized scoring system. This included evaluating the extent of bronchiole injury, capillary congestion, thickening of alveolar septa, protein leakage into alveolar spaces, and inflammatory cell infiltration. Each parameter was scored on a scale from 0 to 3 (0 = No injury, 1 = mild, 2 = Moderate, 3 = Severe), with the total possible score ranging from 0 (no damage) to 15 (maximum damage). The scores were assigned by two independent observers blinded to the experimental groups.

### Western blot

Rat lung tissue was lysed with pre-cooled RIPA lysis buffer, and the supernatant was collected after centrifugation at 12,000 g for 20 min. Protein quantification was performed using the BCA protein quantitation kit (Biyuntian Biotechnology, ShangHai, China). Fifty micrograms of each sample were loaded, and 15 % SDS-PAGE electrophoresis was performed to separate proteins, which were then transferred to a PVDF membrane. The membrane was blocked with 3 % BSA at room temperature for 2 h and then incubated with primary antibodies against NGF (1:500, Bode Biotechnology Co., LTD, WuHan, China), proNGF (1:1000, Abcam, Cambridge, MA, USA), p75^NTR^ (1:500, Bode Biotechnology Co., LTD, WuHan, China), and β-actin (1:1000, Bode Biotechnology Co., LTD, WuHan, China) overnight at 4 °C. After washing the membrane with TBST, it was incubated with secondary antibodies (1:2000, Li-Cor Bioscience, USA) for 2 h. After washing the membrane with TBST, protein blots were viewed using ECL chemiluminescence by Mini-Trans Blot transfer apparatus (Bio-Rad, USA). The relative protein expression was determined by the target band to β-actin density for inter-group comparison.

### Statistical analysis

SPSS 23.0 software was used for statistical analysis. Measurement data were expressed as mean ± SD. One-way analysis of variance was used for the analysis of significant differences, and Tukey's multiple comparison tests were performed for the comparison of multiple groups; *p* < 0.05 was considered statistically significant.

## Results

### Comparison of HE staining results of lung tissue

**Fig. 1:**
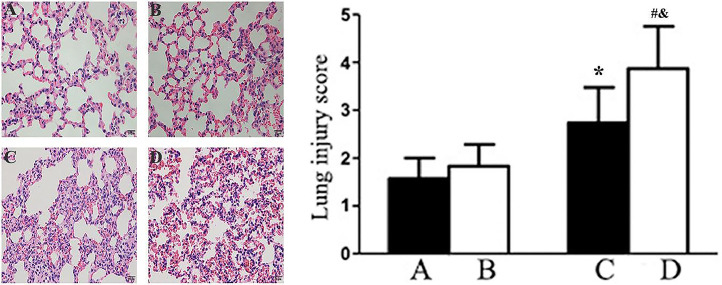
HE staining (×100) of lung tissue and lung injury score. Panels A‒D: Hematoxylin and Eosin (H&E) staining of lung tissues from different experimental groups. Scale bar = 50 µm. Data are expressed as $\bar{x}$± SD. (A) Young Sham: Young rats that underwent sham surgery showing clear alveolar structures with minimal congestion, (B) Elderly-Sham: Elderly rats that underwent sham surgery showing minimal alveolar thickening and slight congestion, (C) Yong-I/R: Young rats that underwent ischemia-reperfusion exhibiting moderate alveolar wall thickening and congestion. (D) Elderly-I/R: Elderly rats that underwent ischemia-reperfusion displaying pronounced alveolar wall thickening, congestion, and significant inflammatory infiltration. Compared with A, *p < 0.05; Compared with B, ^#^*p* < 0.05; Compared with C, ^&^*p* < 0.05.

**Fig. 2:**
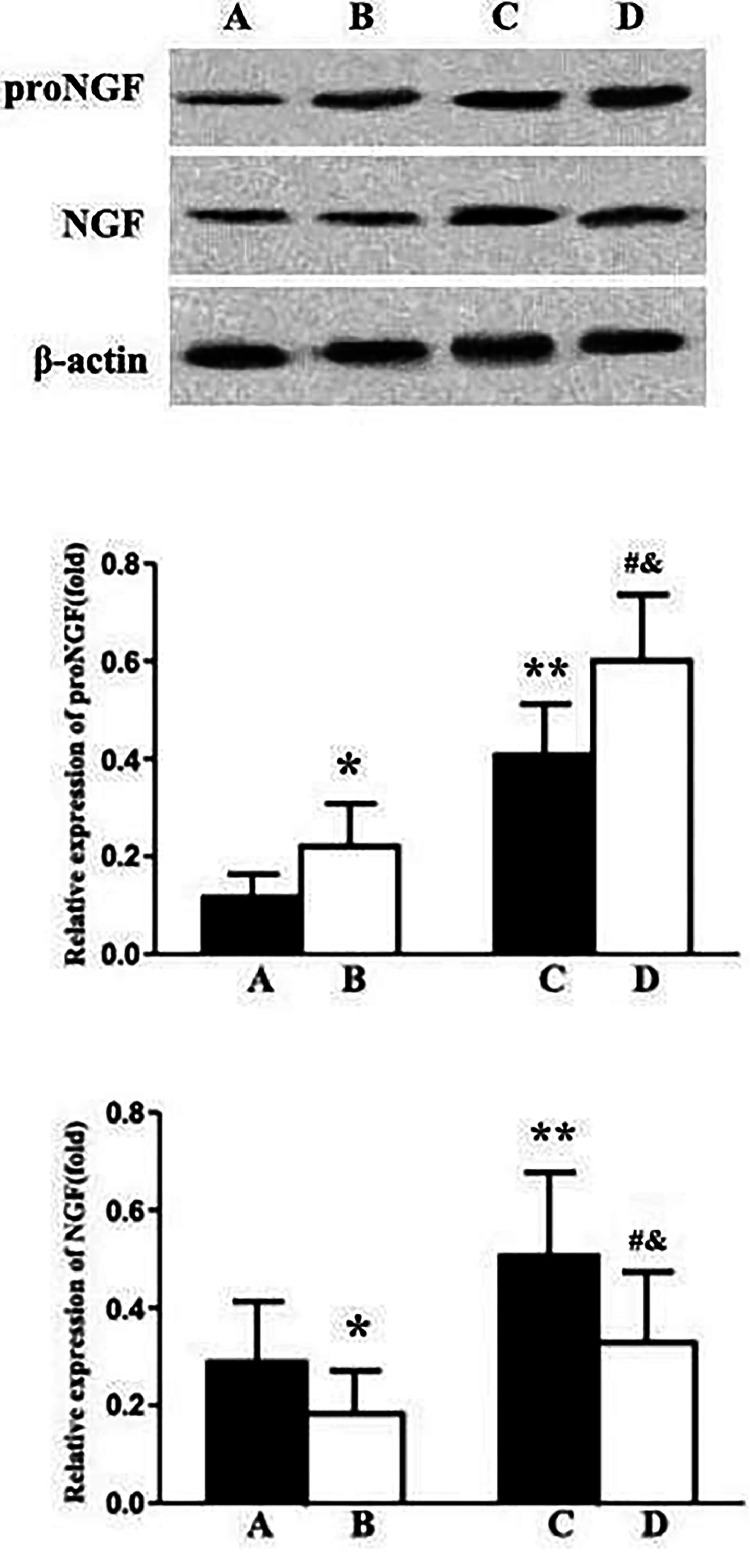
Western Blot Analysis of p75 Neurotrophin Receptor (p75NTR) Expression in Rat Lung Tissues Note: Data are expressed as $\bar{x}$±SD. (A) Yong-Sham, (B) Elderly-Sham, (C) Yong-I/R, (D) Elderly-I/R. Compared with A, *p < 0.05, **p < 0.01; Compared with B, # *p* < 0.05; Compared with C, & *p* < 0.05.

In the histopathological examination of lung tissues, as illustrated in Fig. 1, notable differences were observed across the experimental groups. Panels A through D display hematoxylin and eosin-stained sections of lung tissues from young and elderly rats subjected to either sham surgery or Ischemia-Reperfusion (I/R). Young Sham Group exhibited clear and well-preserved alveolar structures with minimal signs of congestion or inflammation, indicating negligible lung injury. This group served as the baseline control. Elderly Sham Group (Fig. 1B) showed slight thickening of the alveolar septa and minimal congestion, suggesting a slightly increased vulnerability to lung injury with age, even without I/R intervention. Young I/R Group (Fig. 1C) demonstrated moderate alveolar wall thickening and increased capillary congestion, indicative of lung injury due to the I/R procedure. This group had a statistically significant increase in lung injury scores compared to the Young Sham Group (*p* < 0.05), as evidenced by the quantitative scoring graph associated with Fig. 1. The elderly I/R Group (Fig. 1D) exhibited the most severe changes, with pronounced thickening of alveolar walls, significant congestion, and extensive infiltration of inflammatory cells. This group had the highest lung injury score, which was significantly greater than both the corresponding age-matched sham group and the younger I/R group (^#^ *p* < 0.05, ^&^ *p* < 0.05). The quantitative assessment of lung injury scores, depicted in the graph accompanying Fig. 1, clearly illustrates an increase in pathology scores with age and I/R treatment. There was a statistically significant difference between the Elderly-Sham and Young-I/R groups (*p* < 0.05), indicating that even without I/R intervention, elderly rats have a higher baseline level of lung injury compared to young rats subjected to I/R. Similarly, there was a statistically significant difference between the Young-Sham and Elderly-I/R groups (*p* < 0.05), highlighting the severe impact of I/R on elderly rats compared to the baseline condition in young rats. These scores validate the visual differences noted in the histopathological panels and highlight the impact of cerebral ischemia-reperfusion on lung tissue, particularly accentuated in elderly rats. Additionally, the pathological scores of lung tissues in the elderly I/R group were significantly higher than those in the young I/R group (*p* < 0.05). (Fig. 2).

### Comparison of NGF and proNGF protein expression in lung tissue

Compared to the young sham group, there was a significant increase in proNGF protein expression in the lung tissues of elderly sham rats (*p* < 0.05), whereas the expression of NGF decreased (*p* < 0.05). Similarly, compared to their respective sham groups, both young I/R and elderly I/R groups exhibited elevated expression levels of proNGF and NGF proteins in lung tissues (*p* < 0.05). Notably, in the elderly I/R group, the expression of proNGF was higher than that in the young I/R group (*p* < 0.05), while the expression of NGF was lower (*p* < 0.05). The Elderly-Sham group showed significantly higher proNGF and lower NGF levels compared to the Young I/R group (*p* < 0.05), indicating an age-related alteration in neurotrophic factor expression even in the absence of I/R injury. The Elderly I/R group exhibited significantly higher proNGF levels and lower NGF levels compared to the Young Sham group (*p* < 0.05), emphasizing the combined effect of aging and I/R on neurotrophic factor expression (Fig. 2).

### Comparison of p75^NTR^ protein expression in lung tissue

**Fig. 3:**
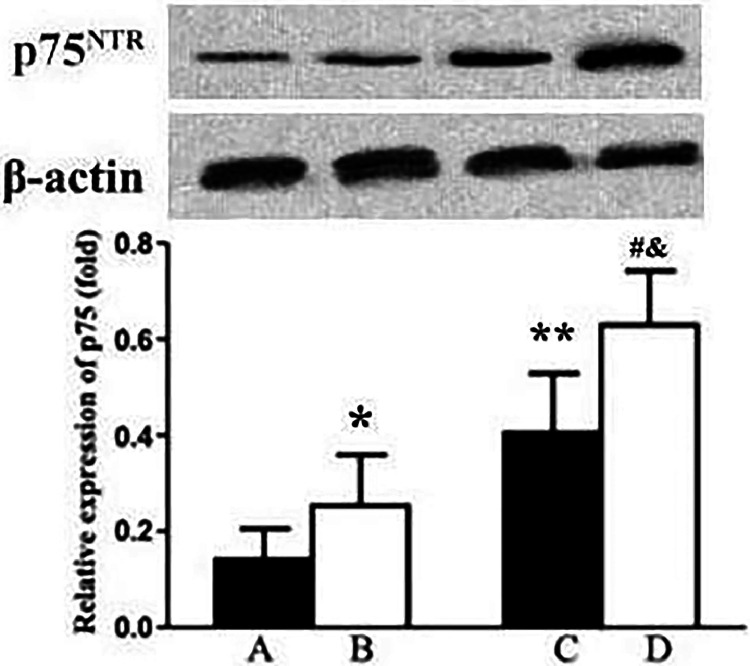
The expression of p75^NTR^ in lung tissues. Data are expressed as $\bar{x}$±SD. (A) Yong-Sham, (B) Elderly-Sham, (C) Yong-I/R, (D) Elderly-I/R. Compared with A, *p < 0.05, ^⁎⁎^*p* < 0.01; Compared with B, ^#^*p* < 0.05; Compared with C, ^&^*p* < 0.05.

In comparison to the young sham group, there was a notable increase in p75^NTR^ protein expression in the lung tissues of elderly sham rats (*p* < 0.05). Similarly, compared to the sham-operated groups of corresponding ages, both the young I/R and elderly I/R groups demonstrated elevated p75^NTR^ protein expression levels in lung tissues (*p* < 0.05). Moreover, in the elderly I/R group, the expression of p75^NTR^ in lung tissues was significantly higher than that in the young I/R group (*p* < 0.05). The Elderly-Sham group had significantly higher p75^NTR^ levels compared to the Young I/R group (*p* < 0.05), suggesting that aging alone increases p75^NTR^ expression. The Elderly I/R group showed significantly higher p75^NTR^ levels compared to the Young Sham group (*p* < 0.05), indicating the combined impact of aging and I/R on p75^NTR^ expression (Fig. 3).

## Discussion

Clinical observations indicate that patients with acute cerebral ischemia frequently develop neurogenic pulmonary edema.^12^ Previous studies have shown that local permanent cerebral ischemia in rats can lead to pulmonary edema and damage to endothelial and epithelial cells.^6^ In this study, the authors observed significant lung pathological changes, such as pulmonary edema and thickening of alveolar septa, in both young and elderly rats after cerebral I/R. The severity of lung injury was greater in elderly rats, as demonstrated by higher pathological scores, indicating a greater vulnerability to I/R-induced lung damage with aging.

NGF, a member of the neurotrophic factor family, plays a crucial role in promoting neuron differentiation and survival.^13^ Its mature form binds to TrkA receptors, stimulating neurite growth and survival pathways, while its precursor form, proNGF, binds to p75^NTR^, triggering apoptotic pathways.^14^ Beyond neural tissues, NGF and its receptors are expressed in various lung cells, including epithelial cells, smooth muscle cells, fibroblasts, and immune cells.^15^ NGF signaling regulates lung tissue development, inflammation, injury, and fibrosis. Previous studies have shown that local tissue damage, inflammation, and cytokines could transiently upregulate NGF expression.^16^ This study also demonstrated that NGF and proNGF expression in lung tissue of young and elderly rats were increased after cerebral I/R. Additionally, compared to the young sham group, NGF expression in lung tissue of elderly sham rats was significantly decreased, while proNGF was increased. These findings further confirmed the effect of aging and injury on NGF/proNGF expression of the lung.^17^

The p75^NTR^ is the receptor for neurotrophic factors and its regulation of cell survival or apoptosis. When TrkA is co-expressed with p75^NTR^, p75^NTR^ can increase the binding affinity of TrkA receptors to NGF.^18^ In the absence of TrkA, proNGF binds to p75^NTR^ and sortilin, inducing cell apoptosis and damage.^19^ Compared with TrkA, the relative expression level of p75^NTR^ may be a key factor in regulating the activity of NGF/proNGF. When PC12 cells were cultured under an increased p75^NTR^/TrkA ratio, PC12 cells were more susceptible to cell death mediated by proNGF.^20^ Beattie et al. .^21^ reported a significant increase in p75^NTR^ expression after spinal cord injury in rats. In this study, the expression of p75^NTR^ in lung tissue of young and elderly rats was increased after cerebral I/R. Additionally, expression of p75^NTR^ in neuronal tissue of aging rodents increases, while TrkA decreases. Small molecule inhibitors of p75^NTR^ binding to neurotrophic factors have shown efficacy in models of Alzheimer's disease and neurodegeneration.^22^ In the present, the expression of p75^NTR^ protein in the lung tissue of elderly rats was higher than that in young rats. Therefore, aging and injury may induce high expression of p75^NTR^, thereby initiating the apoptotic activity of proNGF.

The strengths of this study include the use of well-established animal models to explore age-related differences in the neuroimmune response to cerebral ischemia-reperfusion. The detailed histological and protein expression analyses provide valuable insights into the pathological mechanisms potentially driving increased lung injury in elderly subjects. However, this study has limitations that must be acknowledged. One major limitation is the lack of direct measurement of TrkA, which prevents a comprehensive analysis of neurotrophic signaling dynamics. Additionally, the study was confined to male rats, which may limit the generalizability of the findings to females. Further research involving a broader range of markers and the inclusion of both sexes would enhance the understanding of the mechanisms at play.

## Conclusions

In conclusion, this study elucidates the alternation of NGF, proNGF, and p75^NTR^ expression in lung tissue of aged rats following cerebral I/R, potentially correlating with their susceptibility to age-related lung injury. Modulating the NGF signaling pathway holds promise for mitigating cerebral ischemia-induced lung injury in elderly individuals, offering preliminary insights for the development of targeted therapeutic interventions.

## Ethics approval and consent to participate

Institutional ethical issues: The experimental protocol was approved by the Animal Experimentation Ethics Committee of the Nanjing University of Chinese Medicine. The Ethics Committee study protocol number is 202309A008. Experimental animals underwent all procedures under anesthesia, and every effort was made to minimize their pain, suffering, and death.

## Consent for publication

Not applicable.

### Availability of data and materials

All data generated or analyzed during this study are included in this published article.

## Authors' contributions

CH, DQ conceived of the study, and CJ, TQ and XL participated in its design and data analysis and statistics and WY and GXY helped to draft the manuscript. All authors read and approved the final manuscript.

## Declaration of competing interest

All of the authors had no any personal, financial, commercial, or academic conflicts of interest separately.

## References

[1] Wang Y., Hong F., Yang S (2022). Roles of nitric oxide in brain ischemia and reperfusion. Int J Mol Sci.

[2] Xu Q., Ye Y., Wang Z., Zhu H., Li Y., Wang J. (2022). NLRP3 knockout protects against lung injury induced by cerebral ischemia-reperfusion. Oxid Med Cell Longev.

[3] Wang S.B., Ye Q., Tu J.W., Yu X.Y. (2018). Transient cerebral ischemia/reperfusion-induced acute lung injury in rats associated with protein kinase C alpha expression. Int J Clin Exp Pathol.

[4] Winklewski P.J., Radkowski M., Demkow U. (2014). Cross-talk between the inflammatory response, sympathetic activation and pulmonary infection in the ischemic stroke. J Neuroinflammation.

[5] Fisher A.J., Donnelly S.C., Hirani N., Burdick M.D., Strieter R.M., Dark J.H. (1999). Enhanced pulmonary inflammation in organ donors following fatal non-traumatic brain injury. Lancet.

[6] Samary C.S., Ramos A.B., Maia L.A., Rocha N.N., Santos C.L., Magalhães R.F. (2018). Focal ischemic stroke leads to lung injury and reduces alveolar macrophage phagocytic capability in rats. Crit Care.

[7] Austin V., Ku J.M., Miller A.A., Vlahos R. (2019). Ischaemic stroke in mice induces lung inflammation but not acute lung injury. Sci Rep.

[8] Prakash Y., Thompson M.A., Meuchel L., Pabelick C.M., Mantilla C.B., Zaidi S. (2010). Neurotrophins in lung health and disease. Expert Rev Respir Med.

[9] Capsoni S., Brandi R., Arisi I., D'Onofrio M., Cattaneo A (2011). A dual mechanism linking NGF/proNGF imbalance and early inflammation to Alzheimer's disease neurodegeneration in the AD11 anti-NGF mouse model. CNS Neurol Disord Drug Targets.

[10] Bradshaw R.A., Pundavela J., Biarc J., Chalkley R.J., Burlingame A.L., Hondermarck H. (2015). NGF and ProNGF: regulation of neuronal and neoplastic responses through receptor signaling. Adv Biol Regul.

[11] Tang Y.N., Zhang G.F., Chen H.L., Sun X.P., Qin W.W., Shi F. (2020). Selective brain hypothermia-induced neuroprotection against focal cerebral ischemia/reperfusion injury is associated with Fis1 inhibition. Neural Regen Res.

[12] Liarakos A.L., Tran P. (2022). Delayed onset of neurogenic pulmonary oedema following an evolving ischaemic stroke. BMJ Case Rep.

[13] Sariola H. (2001). The neurotrophic factors in non-neuronal tissues. Cell Mol Life Sci.

[14] Micera A., Vigneti E., Pickholtz D., Reich R., Pappo O., Bonini S. (2001). Nerve growth factor displays stimulatory effects on human skin and lung fibroblasts, demonstrating a direct role for this factor in tissue repair. Proc Natl Acad Sci U S A.

[15] Sonar S.S., Schwinge D., Kilic A., Yildirim A.O., Conrad M.L., Seidler K. (2010). Nerve growth factor enhances Clara cell proliferation after lung injury. Eur Respir J.

[16] Pöyhönen S., Er S., Domanskyi A., Airavaara M. (2019). Effects of neurotrophic factors in glial cells in the central nervous system: expression and properties in neurodegeneration and injury. Front Physiol.

[17] Li Y., Wu F., Zhou M., Zhou J., Cui S., Guo J. (2022). ProNGF/NGF modulates autophagy and apoptosis through PI3K/Akt/mTOR and ERK signaling pathways following cerebral ischemia-reperfusion in Rats. Oxid Med Cell Longev.

[18] Morel L., Domingues O., Zimmer J., Michel T. (2020). Revisiting the role of neurotrophic factors in inflammation. Cells.

[19] Harrington A.W., Leiner B., Blechschmitt C., Arevalo J.C., Lee R., Mörl K. (2004). Secreted proNGF is a pathophysiological death-inducing ligand after adult CNS injury. Proc Natl Acad Sci U S A.

[20] Masoudi R., Ioannou M.S., Coughlin M.D., Pagadala P., Neet K.E., Clewes O. (2009). Biological activity of nerve growth factor precursor is dependent upon relative levels of its receptors. J Biol Chem.

[21] Beattie M.S., Harrington A.W., Lee R., Kim J.Y., Boyce S.L., Longo F.M. (2002). ProNGF induces p75-mediated death of oligodendrocytes following spinal cord injury. Neuron.

[22] Jr Terry AV, A Kutiyanawalla, Pillai A (2011). Age-dependent alterations in nerve growth factor (NGF)-related proteins, sortilin, and learning and memory in rats. Physiol Behav.

